# Extracellular Vesicles–Encapsulated MicroRNA-125b Produced in Genetically Modified Mesenchymal Stromal Cells Inhibits Hepatocellular Carcinoma Cell Proliferation

**DOI:** 10.3390/cells8121560

**Published:** 2019-12-03

**Authors:** Silvia Baldari, Giuliana Di Rocco, Alessandra Magenta, Mario Picozza, Gabriele Toietta

**Affiliations:** 1 Department of Research, Advanced Diagnostic, and Technological Innovation, IRCCS Regina Elena National Cancer Institute, Via E. Chianesi 53, 00144 Rome, Italy; silvia.baldari@ifo.gov.it (S.B.); giuliana.dirocco@ifo.gov.it (G.D.R.); 2 Department of Medical Surgical Sciences and Biotechnologies, University of Rome “La Sapienza”, C.so della Repubblica 79, 04100 Latina, Italy; 3 Istituto Dermopatico dell’Immacolata, IDI-IRCCS, Via dei Monti di Creta 104, 00167 Rome, Italy; ale.magenta@gmail.com; 4 Laboratory of Neuroimmunology, IRCCS Fondazione Santa Lucia, Via del Fosso di Fiorano 64, 00143 Rome, Italy; picozzamario@gmail.com

**Keywords:** hepatocellular carcinoma, microRNAs, extracellular vesicles, cell proliferation, adipose tissue-derived stromal cells

## Abstract

Hepatocellular carcinoma (HCC) is the most frequent type of primary liver cancer and one of the prominent causes of cancer mortality, leading to approximately 780,000 deaths per year worldwide. Down-regulation of microRNA-125b (miR-125b) is a prognostic indicator in HCC patients. Conversely, over-expression of miR-125b in HCC cells induces cell cycle arrest, inhibits proliferation, migration and invasion. Extracellular vesicles (EVs) function as intercellular messengers transferring proteins, RNAs, DNAs, carbohydrates, and lipids. Since EVs protect their cargo from degradation, delivery of therapeutic bioactive molecules, in particular miRNAs, through EVs represents an innovative avenue for cancer therapy. In this study, we evaluated a replacement strategy for the treatment of HCC via delivery of EVs secreted from human adipose tissue-derived mesenchymal stromal/medicinal signaling cells (ASCs) genetically modified with a lentiviral vector expressing miR-125b with a specific ExoMotif sequence tag to enhance the loading into extracellular vesicles. In particular, we determined that the delivery of miR-125b-loaded EVs produced in engineered ASCs specifically reduces HCC cell proliferation in vitro modulating a series of miR-125b targets, which belong to the p53 signaling pathway. This proof-of-concept study supports the development of innovative therapeutic strategies for HCC via EV-mediated miRNA delivery.

## 1. Introduction

Micro RNAs (miRNAs) are a class of approximately 22 nucleotide-long non-coding RNAs predominantly involved in the regulation of target gene expression, mainly at the post transcriptional level [1]. More than 2500 miRNAs have been identified in eukaryotic cells, and they have been shown to play a pivotal role in regulating different physiological and pathological processes including cancer development, metastasis and drug resistance [2]. Argonaute proteins [3] or inclusion into extracellular microvesicles (EVs) [4] protect circulating miRNAs from RNase degradation, allowing for action on distal target cells.

EVs are membranous vesicles originating from several cells via multivesicular bodies (exosomes), shedding from the cell membrane (microparticles), or produced by apoptotic cells (apoptotic bodies), and released in many body fluids [5,6]. The International Society for Extracellular Vesicles recommends the generic term “extracellular vesicles” (EVs) to collectively denote vesicles obtained from biological samples or cell culture conditioned medium, regardless of differences in biogenesis and composition [7]. EVs function as intercellular messengers transferring proteins, RNA (messenger RNA, long noncoding RNA, microRNA), DNA (mitochondrial DNA, chromosomal DNA), carbohydrates, and bioactive lipids. The content of the cargo packaged into EVs may be different from that in the originating cells, indicating that the loading process is selective [8]. Circulating EVs can, therefore, represent powerful, minimally invasive, specific diagnostic, and prognostic biomarkers for several diseases including cancer [9]. On the other hand, delivery of therapeutic bioactive molecules, in particular miRNAs [10], through EVs may be an innovative avenue for cancer therapy [4,11,12,13,14,15]. In facts, as delivery vehicles, EVs support the targeted delivery of microRNAs and small interfering RNAs, protecting them from degradation. Moreover, EVs have negligible toxicity and immunogenicity permitting repeated administration and can cross the blood–brain barrier [16,17], opening the perspective use of EV-mediated delivery for the treatment of neurodegenerative diseases [18].

Hepatocellular carcinoma (HCC) is the sixth most common tumor in the world and the second highest cause of cancer mortality with increasing incidence, especially in developing nations [19]. Surgical resection is the first line treatment, but only a subset of patients is eligible for this approach [20]. Innovative treatments for the management of HCC are therefore needed. The most promising strategy is represented by targeted therapy, aimed at inhibiting molecular pathways critical for tumor growth or in restoring oncosuppressor signals [21]. HCC tumor immune-profile can be influenced by EV-mediated miRNA transfer [22,23]. Dis-regulated expression of several miRNAs has been associated with HCC progression [24,25,26]. In particular, down-regulation of miR-125b has been described as a prognostic indicator in hepatocellular carcinoma patients, as well as in HCC cell lines [27]. Conversely, over-expression of miR-125b in HCC cells induces cell cycle arrest, inhibits cell proliferation, migration and invasion, targeting the oncogenes LIN28B [28], Mcl-1 and IL6R [29], and TXNRD1 [30] sirtuin 6 [31] and 7 [32]. Taken together, these observations suggest that miR-125b acts as a oncosuppressor in HCC [29], albeit its role as oncogene has been described for other kinds of cancers [33].

Mesenchymal stromal/stem cells, also referred as medicinal signaling cells (MSCs) [34], isolated from different sources represent a tool for regenerative medicine and tissue engineering [35]. One of the prominent mechanisms by which they exert their beneficial effect on tissue remodeling is by secretion of EVs containing bioactive molecules [36,37]. Indeed, MSCs represent a proficient source of EVs [38]. Content and nature of miRNAs present in EVs depend on cellular sources. Several reports indicate that miR-125b can be detected in EVs isolated from MSCs [39]. Nonetheless, stoichiometric studies suggest that the amount of a single mRNA present in the majority of EVs is below the minimal level likely to produce a functionally relevant effect in recipient cells [40]. Therefore, finding an effective strategy to engineer EVs in order to increase the therapeutic index of EV-mediated miRNA delivery against HCC remains a major challenge [41,42].

Genetically engineering MSCs in order to overexpress miRNAs containing specific sequences (ExoMotif, GGAG in the 3′ half of the microRNA and C/UCCU/G) to promote target packaging into EVs [43], may represent a suitable strategy to obtain EVs loaded with specific miRNAs, which can be delivered to restore tumor-suppressor activity in HCC. In the current study, we evaluate a miR-125b replacement strategy for the treatment of HCC via delivery of EVs-containing miR-125b produced in engineered human mesenchymal cells isolated from adipose tissue.

## 2. Materials and Methods

### 2.1. Cell Isolation and Culture

Human adipose tissue-derived mesenchymal stromal cells (ASC) [44] were isolated from lipoaspirates collected from Caucasian females (age range 25–56 years) undergoing liposuction, as previously described [45]. All donors gave their written informed consent in accordance with the standards of the University of Rome “La Sapienza” Ethical Committee (Authorization n.1794/15, 13 February 2015) and the principles expressed in the Declaration of Helsinki.

Human hepatocarcinoma Hep G2 (ATCC^®^ Number: HB-8065), human colorectal carcinoma HCT 116 (ATCC^®^ Number: CCL-247), human cutaneous melanoma cells MeWo (ATCC^®^ Number: HTB-65), and human embryonic kidney cells 293T (ATCC^®^ Number: CRL-3216) were cultured following American Type Culture Collection (ATCC) recommendations. Characteristics and culture conditions for human HuH-7 [46] and murine BW1J [47] hepatoma cells have been previously described.

### 2.2. Viral Vectors Production and Gene Transfer

The recombinant vesicular stomatitis virus-pseudotyped lentiviral vector XMIRXpress miR125-5p (System Biosciences, Mountain View, CA, USA), the LV-XPack GFP vector, a green fluorescent protein (GFP)-expressing lentiviral vector derived from the XPack-CMV-XP-MCS-EF1-Puro vector (System Biosciences) were produced according to standard protocols [48]. Both lentiviral vectors contain the previously characterized ExoMotif (XPack tag) which directs the gene product to be exported into secreted EVs [49]. Lentiviral vectors at approximate multiplicity of infection 10, in presence of 8 µg/mL polybrene (Sigma-Aldrich, St. Louis, MO, USA), were used to transduce ASC. Seventy-two hours after transduction, the cells were incubated in DMEM containing 10% FBS and 1 µg/mL puromycin for seven days. The puromycin resistant cells were called ASC-EVmiR125b or ASC-EVmirCRL.

### 2.3. Extracellular Vesicles Isolation

For EV collection, human ASCs were transduced with either the LV XMIRXpress miR-125-5p or the LV-XPack GFP vector, LV XMIRXpress miR-224-5p as control. Efficiency of transduction was estimated to be above 85% by green fluorescence analysis performed by the ZOE Fluorescent Cell Imager (Bio-Rad Laboratories, Hercules, CA, USA). Transduced cells were cultured in Dulbecco’s minimal essential medium (DMEM), 100 U/mL penicillin, 100 µg/mL streptomycin, supplemented with exosome-depleted fetal bovine serum (Exo FBS, System Biosciences) for 48 h at 37 °C in 5% CO_2_, then the conditioned medium was collected. Medium was centrifuged at 3000× *g* for 5 min, filtered using 0.2 micron low-protein-binding filter, and then concentrated using an Amicon Ultra filter with nominal molecular weight limit (NMWL) 100 kD (Millipore, Darmstadt, Germany). Purification of EVs from the concentrated medium was performed using the ExoQuick reagent (System Biosciences), according to manufacturer’s specifications.

### 2.4. Fluorescence Microscopy Analysis

Human ASCs stably expressing EV miR-125b and Hep G2 cells treated with 90 µg of miR-125b purified EVs, were seeded, respectively, on glass slides and into 12-well plates (1 × 10^4^ cells/well). For the analysis, which was performed at the same time point of the other functional assays, cells were rinsed with phosphate-buffered saline (PBS) and fixed for 10 min at room temperature with 2% paraformaldehyde followed by permeabilization with 0.4% Triton X-100 in PBS. Nuclei were counterstained with Hoechst. The cells were examined by confocal fluorescence microscopy (Zeiss LSM 880 Axio Observer, Jena, Germany).

### 2.5. Immunoblot Analysis

Protein content was measured using the Bradford assay. Protein lysates were subjected, under non reducing conditions, to 10% sodium dodecyl sulfate-polyacrylamide gel electrophoresis and transferred on nitrocellulose membranes for Western blot analysis using antibodies against CD63 (ThermoFisher Scientific, Waltham, MA, USA), p53 (Santa Cruz Biotechnology, Santa Cruz, CA, USA) and glyceraldehyde phosphate dehydrogenase (GAPDH) as protein loading control. Densitometric quantification of the immunoblot bands was performed using the ImageJ software (National Institutes of Health, Bethesda, MD, USA).

### 2.6. Quantitative Real-Time Polymerase Chain Reaction

Total RNA was extracted from the EV preparations. TaqMan probe for miR-125b (hsa-miR-125b #00049, ThermoFisher Scientific) was used for qRT-PCR quantification on ABI PRISM 7900 Sequence Detection System (ThermoFisher Scientific). miR-125b relative expression was normalized to *Caenorhabditis elegans* miRNA (Cel-miR-39) (ThermoFisher Scientific), as previously described [50].

### 2.7. In Vitro Cell Proliferation Assay

Cell proliferation was measured using the WST-1 cell proliferation assay kit (Takara, Clontech, Mountain View, CA, USA), according to manufacturer’s instructions. Moreover, cell proliferation was also measured using a label-free, non-invasive cellular confluence assay using the IncuCyte Live-Cell Imaging Systems (Essen Bioscience, Ann Arbor, MI, USA). In particular, Hep G2 cells (1 × 10^3^ cells/well) were seeded on a 96-well plate in triplicate and phase contrast images were taken using the IncuCyte™ at 24 h intervals for seven days. Cell confluence data were analyzed using the IncuCyte™ (S3 Live-Cell Analysis System software (v2019B)).

### 2.8. Colony Formation Assay

Cells were plated at a density of 7.0 × 10^3^/60-mm tissue culture dish and then cultured in a humidified CO_2_ incubator (5% CO_2_/95% air) at 37 °C. The medium was changed every 3–4 days. On day 7, cells were stained with crystal violet and observed under an inverted microscope. The numbers of colonies in each plate were counted and colony area quantified using the ImageJ software [51].

### 2.9. Cytofluorimetric Analysis

Flow cytometry analysis of EV preparations was performed with a CytoFLEX cell analyzer (Beckman Coulter, Brea, CA, USA) as previously described [52] with slight modifications. Briefly, 15 µL of purified EV suspensions were stained in 45 µL final volume with optimal dilutions of CD81 APC clone JS64 and CD63 PE clone CLBGran/12. Relevant isotype antibodies were used at the same dilutions to ensure specific staining of EV and to evaluate background fluorescence, which served also to set threshold triggering on the CD81 APC channel [53]. Instrument calibration was performed by running Apogee beads (Apogee Flow Systems Ltd., Hertfordshire, UK) with the same instrument settings. All antibodies were from Beckman Coulter.

### 2.10. Human p53 Signaling Pathway Expression Array (RT2 PCR Profiler Array)

Hep G2 cells (1.0 × 10^4^ cells/well), treated with EV purified from conditioned medium from ASCs or ASCs engineered with ExoMotif-tagged microRNA-125b, were collected after 144 h of culture and total RNA was extracted using the RNeasy Mini Kit (Qiagen, Hilden, Germany). cDNA was synthesized using the RT2 first strand kit (Qiagen) from 1000 ng total RNA and qPCR reactions performed using RT2 SYBR^®^ Green qPCR Mastermix (Qiagen) according to the manufacturer’s instructions. An RT2 Profiler PCR array specific for human p53 signaling pathway (Qiagen, Cat. no. PAHS-027ZA) was used to assess the transcriptional levels of 84 different genes using the ABI 7500 Real-Time PCR System (Applied Biosystems). Raw cycle threshold (Ct) values were analyzed using the web-based GeneGlobe Qiagen PCR Array Data Analysis software (https://geneglobe.qiagen.com/, accessed September, 2019). Ct values were normalized based on an automatic selection from housekeeping genes (HKGs) panel of reference genes. Fold change and fold regulation were calculated using delta delta Ct method, with delta Ct calculated between gene of interest (GOI) and an average of HKGs, followed by delta-delta Ct calculations. Fold Change (FC) is obtained using 2∧ (-delta delta Ct) formula, dividing the normalized gene expression [2∧ (-Delta Ct)] in the EV miR-125b samples by the normalized gene expression [2∧ (-Delta CT)] of the Ctrl samples.

### 2.11. Statistical Analyses

Comparison between groups was performed with the INSTAT software (GraphPad, San Diego, CA, USA) using a two-tailed Student’s *t*-test for unpaired data; statistical significance level was set at *p* ≤ 0.05.

## 3. Results

### 3.1. Lentiviral Vector-Mediated ExoMotif-Tagged MicroRNA-125b Transfer Specifically Reduces Proliferation and Survival of Hepatocellular Carcinoma Cells

**Figure 1:**
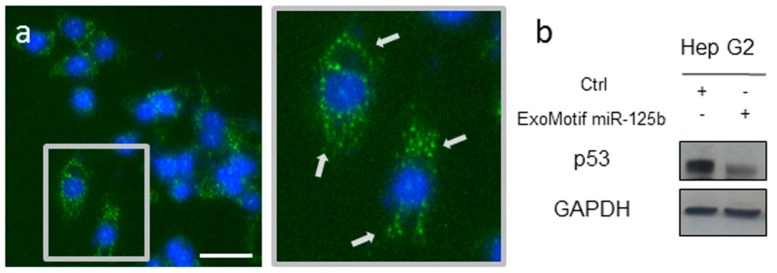
Lentiviral vector-mediated ExoMotif-tagged microRNA-125b gene transfer modulates endogenous p53. (**a**) Fluorescent micrograph of Hep G2 cells transduced with a lentiviral vector expressing miR-125b and green fluorescent protein (GFP) with a specific ExoMotif sequence tag. Nuclei are visualized by Hoechst dye (blue). Inset: Higher magnification view of the boxed area showing transduced cells. Arrows indicate punctate pattern of distribution of GFP. Size bar: 35 μm. (**b**) After 72 h, total cell lysates were obtained and immunoblot analysis for p53, a known miR-125b target, was performed. GAPDH protein level was used as loading control. The plus (+) and minus (−) symbols indicate that cells were transduced or not with the listed vectors, respectively.

Lentiviral vectors expressing either GFP or miR-125b and GFP including a specific ExoMotif sequence tag to target the gene product into extracellular vesicles were produced. Then we transduced human HCC Hep G2 cells by the lentiviral vectors to determine the effect of ExoMotif tagged miR-125b expression. Transduction efficiency was monitored by fluorescence microscopy analysis of GFP expression (Figure 1a). The punctate pattern of localization of GFP expression was consistent with multivesicular bodies targeting. A functional effect of ExoMotif-tagged miRNA-125b gene transfer into Hep G2 cells was determined by assessing the expression of p53 (Figure 1b), known as one direct target of miR-125b [54].

**Figure 2:**
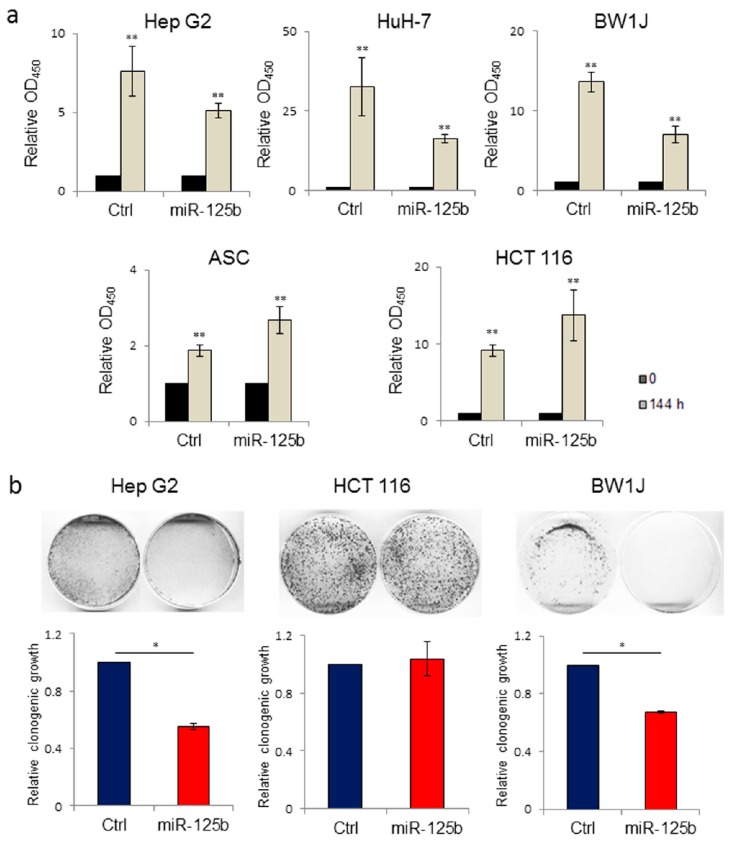
Lentiviral-mediated ExoMotif-tagged microRNA-125b gene transfer affects proliferation and survival of both human and murine hepatocellular carcinoma cell lines. (**a**) Cell proliferation WST-1-based assay performed on human hepatocellular carcinoma cell lines Hep G2, HuH-7 and mouse hepatoma BW1J, human adipose tissue derived stromal cells (ASCs), and human colorectal cancer cells HCT 116 transduced either with a control vector or with a lentiviral vector expressing the ExoMotif tagged miR-125b and GFP. The grey bars represent the proliferation rate of both miR-125b and Ctrl quantified 144 h after transduction and normalized with respect to the time zero-value (T 0) of the corresponding cell line, represented in black bars, set to 1 as reference point. Significance for Ctrl and miR-125b cells is referred to corresponding T 0. ** *p* < 0.001. (**b**) Long-term responses to ExoMotif tagged miR-125b gene transfer was evaluated by clonogenic assay on Hep G2, BW1J, and HCT 116 cell lines. Representative images are shown. The corresponding column graphs report densitometric analyses performed after crystal violet staining. Results are indicated as mean ± standard error of the mean (S.E.M.) of three independent experiments. Significance was assessed by Student’s *t*-test. * *p* < 0.005 respect to control.

MicroRNA-125b is associated with tumor suppressive signaling in several types of cancers [33]. In particular, miR-125b has been described as a tumor suppressor in hepatocellular carcinoma cell lines [29]. We evaluated the effect on cell proliferation of lentiviral-mediated gene transfer into the human HCC cell line Hep G2 of a form of miR-125b containing an ExoMotif tag for miRNA loading into EVs (Figure 2). By cell proliferation assay (Figure 2a) and colony formation analysis (Figure 2b) we determined that ExoMotif tagged miR-125b gene transfer affects proliferation and survival of Hep G2 cells. Similar results were confirmed on the human HCC cell line HuH-7 and on the murine hepatoma cells BW1J (Figure 2a). Conversely, in colon cancer and in some hematopoietic tumors, miR-125b is overexpressed, thus displaying an oncogenic potential that leads to an increase in cell proliferation and inhibition of the apoptotic pathway [33,55]. Accordingly, in contrast to Hep G2 cells, we determined that ExoMotif tagged miR-125b gene transfer into the human colorectal carcinoma HCT 116 cells promotes proliferation (Figure 2a). In addition, we revealed a pro-proliferative effect of miR-125b overexpression in non-tumorigenic human adipose tissue derived mesenchymal stromal cells (ASC) (Figure 2a). Taken together, these results indicate that overexpression by lentiviral-mediated gene transfer of an ExoMotif tagged form of miR-125b specifically reduces proliferation and survival of hepatocellular carcinoma cell lines of human and murine origin.

### 3.2. Human Adipose Tissue Derived Stromal Cells as Source of Extracellular Vesicles Loaded with a ExoMotif-Tagged MicroRNA-125b

**Figure 3:**
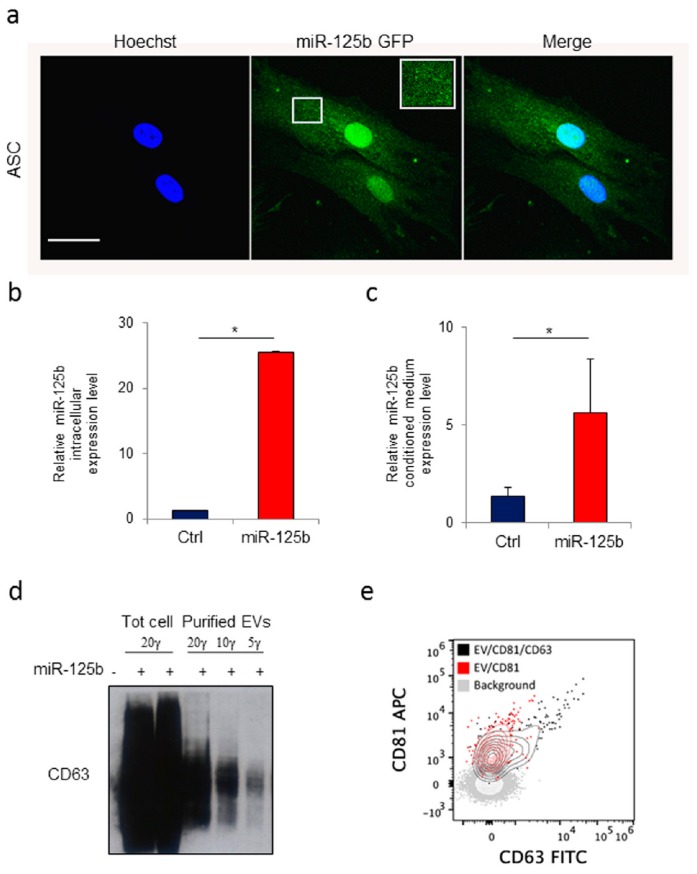
Human adipose tissue derived stromal cells as source of extracellular vesicles loaded with a ExoMotif-tagged microRNA-125b. Human adipose tissue–derived mesenchymal stromal cells (ASCs) were transduced with a lentiviral vector expressing ExoMotif-tagged miR-125b. (**a**) Confocal fluorescence microscopy analysis performed on genetically modified ASCs showing a dotted pattern of expression. Green fluorescence indicates ExoMotif-tagged miR-125b GFP expression; blue staining (Hoechst-staining) indicates cell nuclei. Inset: Higher magnification view of the boxed area. Scale bar = 50 μm. RT-PCR evaluation expression levels of miR-125b in both (**b**) conditioned medium and (**c**) cell lysate collected 48 h after gene transfer with the control (−) or the ExoMotif-tagged miRNA-125b-expressing (+) vectors. Significance was assessed by Student’s *t*-test, * *p* < 0.005. (**d**) Immunoblot with antibodies against the exosomal marker CD63 performed on EV released by genetically modified ASCs purified from the culture medium using the ExoQuick kit. (**e**) Cytofluorimeric analysis of purified EV preparations, stained either with CD81 alone or in combination with CD63 FITC, acquired on a Cytoflex flow cytometer after setting a fluorescence threshold on CD81 signal based on background staining. The plus (+) symbols indicate that cells were transduced or not with the listed vectors, respectively.

Primary and immortalized mesenchymal stromal cells have been described as a suitable source for production and engineering of EVs [38]. To obtain a dependable source of EVs we isolated stromal cells from human adipose tissue collected by non-invasive liposuction as previously described [45]. In order to direct the gene product into EVs, human ASCs were then transduced with lentiviral vectors expressing either GFP or miR-125b and GFP which include a specific ExoMotif sequence [49]. In particular, 1.0 × 10^6^ ASC/100 mm dishes were transduced at multiplicity of infection approximately 10 achieving transduction efficiency above 85%. Fluorescence microscopy analysis of GFP expression revealed the typical dotted pattern along the cell membrane resembling multivesicular bodies (Figure 3a). After 48 h conditioned medium was collected and EVs purified using the ExoQuick kit. RT-PCR analysis for miR-125b expression was performed, confirming specific production of miR-125b by transduced cells (Figure 3b) and presence in cell conditioned medium (Figure 3c). Protein content in transduced cells and in purified EVs preparations was determined and lysates were resolved by SDS–polyacrylamide gel electrophoresis in non-reducing conditions. EV purification was monitored by expression of the member of the tetraspanin family CD63 membrane protein which is abundantly expressed on most human extracellular derived vesicles (Figure 3d). Fluorescence-activated cell sorting (FACS) analysis performed on purified preparation of EVs confirmed the presence of CD63+ vesicles (Figure 3e).

### 3.3. Conditioned Medium from Genetically Modified Adipose Tissue Derived Stromal Cells Expressing ExoMotif-Tagged MicroRNA-125b Specifically Reduces Proliferation of Hepatocellular Carcinoma Cells

**Figure 4:**
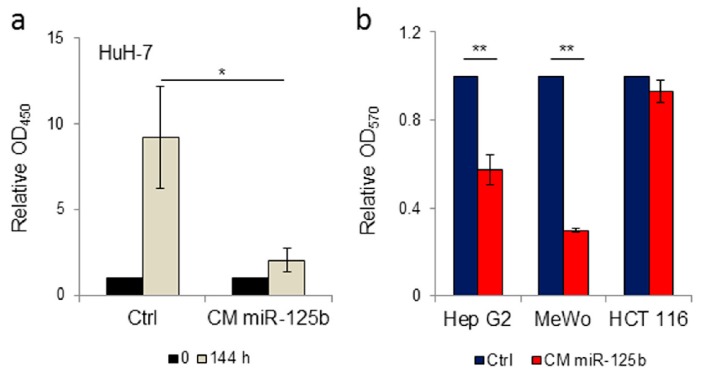
Conditioned medium from ExoMotif-tagged microRNA-125b genetically modified adipose tissue derived stromal cells specifically reduces proliferation of hepatocellular carcinoma cells. (**a**) Cell proliferation assay performed on human HCC cells HuH-7 in presence of conditioned medium collected from human ASC cells transduced either with control or ExoMotif-tagged miR-125b-expressing lentiviral vector. WST-1 assay was performed after 0 (black bars) and 144 h (grey bars) of culture. (**b**) Colony formation assay performed on human hepatocarcinoma Hep G2, melanoma MeWo and colon carcinoma HCT 116 cells cultivated in presence of conditioned medium collected from human ASCs genetically modified with the ExoMotif-tagged miR-125b-expressing lentiviral vector. Cells were seeded at a density of 1 × 10^4^ cells/24-well plates and cultured in freshly prepared conditioned medium every four days. After 10 days of culture cell proliferation assay was performed by crystal violet staining and quantified, upon extraction with de-staining solution, by recording absorbance at 570 nm. Significance was assessed by Student’s *t*-test: * *p* < 0.005, ** *p* < 0.001.

By cell proliferation assay we determined that HuH-7 hepatoma cells maintained in conditioned medium obtained from ExoMotif-tagged miR-125b-expressing ASCs have reduced growth compared to cells cultured in conditioned medium collected from ASC cells genetically transduced with a lentiviral control vector (Figure 4a). Moreover, by colony formation assay we assessed that cultivation in conditioned medium from ExoMotif-tagged miR-125b expressing ASCs determines a reduction of the colony forming capacity in human HCC Hep G2 cells but not in human colon carcinoma HCT 116 cells (Figure 4b). As an additional control, we included human melanoma MeWo cells, in which miR-125b is down-regulated [56]. Accordingly, supplementation of ExoMotif-tagged miR-125b containing medium reduced melanoma MeWo cells proliferation (Figure 4b). These results suggest that engineered ASCs can release functional miR-125b in the conditioned medium, which results in an anti-proliferative effect on HCC cells.

### 3.4. Delivery of EV Isolated from Genetically Modified Adipose Tissue Derived Stromal Cells Expressing ExoMotif-Tagged microRNA-125b Specifically Reduces Proliferation of Hepatocellular Carcinoma Cells

**Figure 5:**
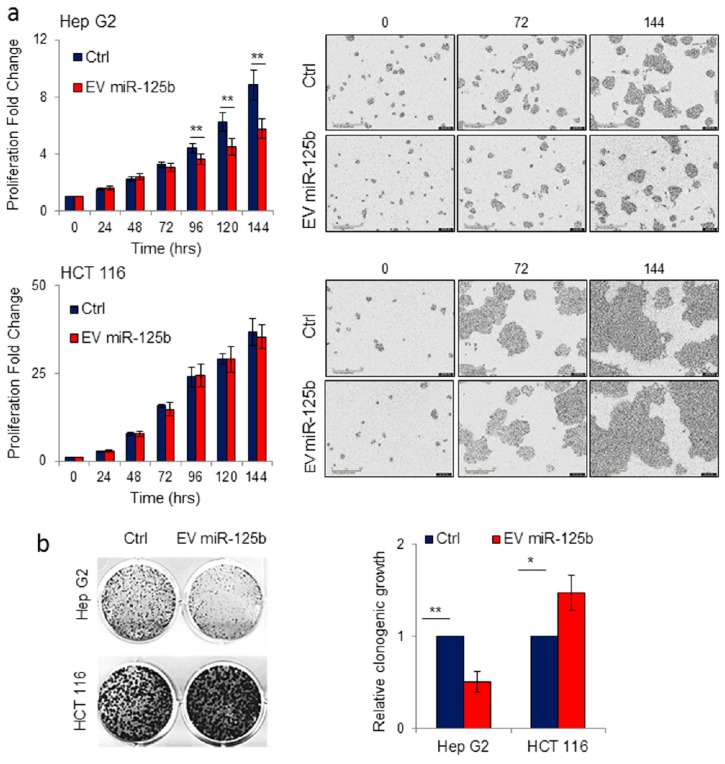
Delivery of EVs isolated from adipose tissue derived stromal cells genetically modified with ExoMotif-tagged microRNA-125b specifically reduces proliferation of hepatocellular carcinoma cells. (**a**) Hep G2 and HCT 116 cell lines were treated with purified EV (10 µg) produced by human ASC cells transduced either with lentiviral control vector (Ctrl) or ExoMotif-tagged microRNA-125b. The effect of EV-mediated miR-125b delivery on target cell proliferation was monitored and quantified by the IncuCyte^®^ live cell imager. Representative images taken using a 10× objective lens at the indicated time points are reported. (**b**) Long-term effect of EV-mediated miR-125b delivery was evaluated by clonogenic assay on Hep G2 and HCT 116 cell lines. After seven days, grown colonies were stained with crystal violet and representative images are shown; the corresponding column graphs report densitometric analyses performed after crystal violet staining. Significance was assessed by Student’s *t*-test. * *p* < 0.05; ** *p* < 0.005 respect to controls.

To specifically evaluate the possibility of using engineered ASC-derived EVs as delivery vehicle of miR-125b, we performed isolation of EVs from conditioned medium obtained from ASCs genetically modified with the lentiviral vector containing the ExoMotif-tagged miR-125b. Supplementation of increasing amount of purified EV preparations determined a reduction of Hep G2 cell proliferation (data not shown). To better investigate the potential of miR-125b-containing EVs to reduce HCC cell proliferation rate we used the IncuCyte™ live-cell imaging system, which allows for automated analysis of the kinetic of cell behavior over time. Cells were cultured and treated with 10 µg of engineered EV purified from ExoMotif-tagged miR-125b-expressing ASCs and observed for seven consecutive days. As reported in Figure 5a, Hep G2 cells showed a significant reduction in cell proliferation starting from 72 h of treatment, while no effect was observed in colon cancer HCT 116 cells. This result was confirmed by metabolic cell viability assay, performed by the WST-1 cell proliferation assay kit.

The long-term effect on Hep G2 cells of the delivery of engineered miR-125b EV purified from ASCs was also monitored by colony formation assay. Hep G2 treated with EV miR-125b presented a significant reduction in colony formation compared to control cells incubated in presence of EVs from non-engineered ASCs. Conversely, no detrimental effect on cell proliferation was observed in colon cancer cell line HCT 116 maintained in the same conditions for seven days (Figure 5b). These results demonstrated that delivery of EVs isolated from ASCs specifically engineered to produce miR-125b loaded EVs, impact hepatocarcinoma cell proliferation.

### 3.5. Effect of MiR-125 Delivery Mediated by Engineered EVs on p53 Signaling

**Figure 6:**
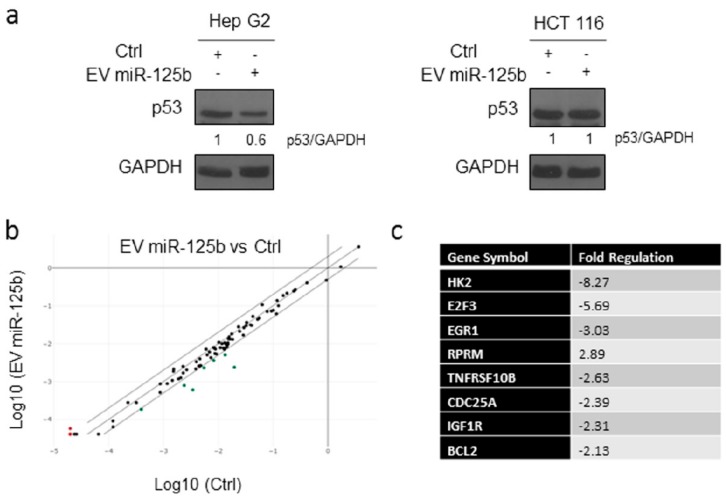
Delivery of EV isolated from adipose tissue derived stromal cells genetically modified with ExoMotif-tagged microRNA-125b affects p53 expression. (**a**) Immunoblot analysis for p53 performed on Hep G2 and HCT 116 cells cultured in presence (+) of miRNA-125b containing or control (−) EVs for 144 h; GAPDH protein level was used as loading control. Densitometry analysis performed using ImageJ software is indicated. (**b**) Analysis of the p53 signaling pathway using a specific RT2 Profiler PCR Array. The expression analysis of 84 genes in EV miR-125b group and Ctrl group was performed. Scatter plot comparison shows gene expression changes in the two groups. The red and green dots represent up-regulated and down-regulated genes, respectively. (**c**) Table reporting the genes changed at least a two-fold differential expression in EV miR-125b group against the control group.

MicroRNA-125b has been proposed as a novel negative regulator of p53 [54]. In this context, to better understand at molecular level the impact of miR-125b delivered through EV, we performed Western blot analysis of p53 levels in Hep G2 and HCT 116 cells treated with EVs isolated from conditioned media from ASCs genetically modified with the lentiviral vector containing the ExoMotif-tagged miR-125b, with respect to control cells in both cell lines. We observed a clear reduction in p53 expression in Hep G2 treated with miR-125b-EVs while no differences were evidenced in the colon cancer cell line HCT 116 undergoing the same treatment, confirming the specific effect of miR-125b in reducing cell proliferation in HCC cells (Figure 6a). Furthermore, we investigated the effect of EV miR-125b delivery on Hep G2 cells by analysis of the p53 signaling pathway using a specific RT2 Profiler PCR Array. Differential expression of several genes was assessed (Figure 6b,c). In particular, several target genes, including hexokinase 2 [57], E2F transcription factor 3 [58], insulin-like growth factor I [59], and B-cell lymphoma 2 [32] were down-regulated while the expression of RPRM (Reprimo, TP53 Dependent G2 Arrest Mediator Homolog) was induced, as evidenced by the scatter plot. The observed deregulation of gene expression was consistent with previous findings describing miR-125b expression in different types of cancer and with miRNA target prediction indicated in the miRDB online database [33].

## 4. Discussion

In the current manuscript, we describe the generation of a lentiviral vector for the expression of miR-125b including a specific sequence (ExoMotif) for loading into EVs, the transduction of human mesenchymal cells and the evaluation of this strategy to produce miR-125b-loaded EVs for reducing HCC cell proliferation. MicroRNA-125b is ubiquitously expressed and its role depends on cellular context. The functional importance of miR-125b dysregulation in several cancers has been extensively evaluated indicating that miR-125b may have oncogenic or tumor-suppressive functions, depending on the tumor type [32]. This paradoxical effect can be explained by the fact that, according to the experimentally validated microRNA-target interactions database (miRTarBase), miR-125b has more than 440 target genes, some of which may oppositely work as tumor suppressors or oncogenes, acting on cell proliferation, apoptosis, epithelial-mesenchymal transition, and tumor invasion and metastasis [60]. Aberrant expression of several miRNAs in the liver has been associated with the development of hepatocellular carcinoma and correlates with its severity and poor prognosis [25]. Consequently, miRNA replacement therapy aiming at restoring expression level of miRNAs with tumor suppressor functions represents an innovative anti HCC therapeutic strategy [61]. To this end, a non-toxic and efficient method for the delivery of the therapeutic miRNA to the liver is needed. EVs have recently attracted considerable attention as an ideal miRNA delivery platform for therapeutic intervention of liver disorders, including hepatocellular carcinoma, due to their extremely low immunogenicity and toxicity profiles and to their ability to increase miRNAs stability in circulation [11,62]. In particular, transfer of miRNA by EVs isolated from mesenchymal cells have been shown to reduce liver fibrosis, promote hepatic regeneration after partial hepatectomy, modulate inflammatory response, and promote anti-tumor activity against hepatocellular carcinoma [4,63].

Engineered EVs with defined miRNA content can be produced ex vivo to serve as biological therapeutic shuttles enabling targeted treatment [64]. Two major methods for miRNA loading into EVs have been developed: (i) exogenous, also referred as chemical- or post-loading methods, by which miRNAs are loaded into purified preparations of EVs, and (ii) endogenous methods, also referred as active- or pre-loading, where miRNAs are loaded by genetically modified donor cells prior to EV shedding [11]. Procedures for miRNAs post-loading include passive diffusion, membrane permeabilization or electroporation which suffer for poor loading efficiency and capacity, possible EV structural damage promoted by the permeabilizing reagents and by induction of EV aggregates due to exposure to electrical fields [65,66]. Conversely, endogenous loading methods exploit the cellular machinery to load the genetic material into EVs during their biogenesis [67]. For instance a method for obtaining miR-199a loaded EV was described by stable transfection of HEK293T cells, yielding to 340 EV/miR-199a copy [68].

Mesenchymal stromal cells exert their regenerative action mainly by paracrine secretion of growth factors, chemokines, and cytokines and EVs; consequently, mesenchymal stromal cells are a promising source for production and engineering of EVs for therapeutic purposes. In particular, antitumor response against HCC has been observed upon administration of EVs isolated from non-genetically modified stromal cells isolated from adipose tissue [69]; anti-fibrotic effect in the liver and increased sensitivity to chemotherapy of HCC cells has been described upon delivery of EV-containing miR-122 produced in mesenchymal cells genetically modified to overexpress miR-122 [70,71]. Similarly, EVs derived from miR-181-5p-modified mesenchymal stromal cells have been associated to reduction of liver fibrosis [72].

We show that lentiviral-mediated miR-125b gene transfer was effective and did not induce significant detrimental effect on ASCs that produced functionally active, mature miR-125b endogenously encapsulated into EVs. The procedure has the potential for flexible large-scale, GMP-compliant production to obtain suitable amounts of purified EVs for clinical applications [73]. Interestingly, delivery of EVs containing enriched content of active miR-125b was able to modify the miRNAs target genes in recipient HCC cells, leading to reduced cell proliferation measured by independent assays, namely WST-1 assay, colony formation analysis and by automated assessment of cell growth changes over time. Among the gene differentially modulated by engineered EV-containing miR-125b produced by mesenchymal cells genetically modified to overexpress miR-125b we detected HX2 (hexokinase 2), which has been described as a miR-125b target in hepatocellular carcinoma [57], the transcription factor E2F3 and insulin-like growth factor I, whose expression has been found deregulated by miR-125b in bladder cancer [58,59], the anti-apoptotic protein Bcl-2, as previously evidenced in HCC [32], as well as CDC25A, a key mediator of cell cycle progression. Our results are therefore consistent with previously reported data indicating the involvement of miR-125b in HCC and in other cancer types.

The aim of our study was mainly to evaluate the feasibility of lentiviral-mediated gene transfer into ASCs to produce engineered EVs. One concern about our findings is that we relied on commercially available reagents developed to isolate EVs from ASC culture medium. Although widely accepted, this method may suffer from some limitations due to possible presence in the EV preparations of a mixed population of vesicles or contaminations of non-EV protein or protein aggregates [74]. As a matter of fact, the development of protocols for EV isolation, the requirements for EV analysis [7,75] and for characterization of EV-associated small non coding RNAs [76] are still ongoing.

Albeit promising, there are several limitations to the implementation of EV-mediated miRNA replacement therapeutic approach including: The need to develop improved and standardized systems for scalable production, isolation and characterization of EVs suitable for clinical translation studies [77,78]; to overcome current problems in order to precisely determine the RNA content into EVs [76,79]; to accurately define dosage and route of administration, since the majority of systemically injected EVs are delivered into the liver and to a lesser extent into spleen, intestine, lungs, pancreas and kidney [37]; and to explore the possibility of targeted delivery of engineered EVs. Moreover, regulatory and safety regulation for EV-based therapeutics in clinical trials need to be fully defined [80].

## 5. Conclusions

Collectively, our results reinforce the possibility to genetically modify mesenchymal cells in order to produce engineered EVs that can deliver their cargo for therapeutic purposes. Moreover, the results confirm the anti-proliferative role of miR-125b in HCC supporting EV-mediated miR-125b delivery as a possible therapeutic strategy.
